# *Variovorax* sp. Has an Optimum Cell Density to Fully Function as a Plant Growth Promoter

**DOI:** 10.3390/microorganisms7030082

**Published:** 2019-03-15

**Authors:** Oyungerel Natsagdorj, Hisayo Sakamoto, Dennis Marvin O. Santiago, Christine D. Santiago, Yoshitake Orikasa, Kazuyuki Okazaki, Seishi Ikeda, Takuji Ohwada

**Affiliations:** 1 United Graduate School of Agricultural Sciences, Iwate University, 18-8 Ueda-sanchome, Morioka, Iwate 020-8550, Japan; oyungereln@ymail.com (O.N.); yosori@obihiro.ac.jp (Y.O.); 2 Department of Life and Food Science, Obihiro University of Agriculture and Veterinary Medicine, Inada-cho, Obihiro, Hokkaido 080-8555, Japan; hisayosakamoto.com@gmail.com; 3 Institute of Food Science and Technology, College of Agriculture and Food Science, University of the Philippines Los Baños, Laguna 4031, Philippines; dosantiago1@up.edu.ph; 4 Department of Science and Technology Philippines-Philippine Council for Agriculture, Aquatic and Natural Resources Research and Development, Los Baños, Laguna 4030, Philippines; christinesantiago_1017@yahoo.com; 5 Hokkaido Agricultural Research Center, National Agriculture and Food Research Organization, 9-4 Shinsei-minami, Memuro-cho, Kasai-gun, Hokkaido 082-0081, Japan; okakazu@affrc.go.jp (K.O.); sikeda67@affrc.go.jp (S.I.)

**Keywords:** plant growth-promoting bacteria, *Variovorax*, bioinoculant, GUS staining

## Abstract

Utilization of plant growth-promoting bacteria colonizing roots is environmentally friendly technology instead of using chemicals in agriculture, and understanding of the effects of their colonization modes in promoting plant growth is important for sustainable agriculture. We herein screened the six potential plant growth-promoting bacteria isolated from *Beta vulgaris* L. (*Rhizobium* sp. HRRK 005, *Polaromonas* sp. HRRK 103, *Variovorax* sp. HRRK 170, *Mesorhizobium* sp. HRRK 190, *Streptomyces* sp. HRTK 192, and *Novosphingobium* sp. HRRK 193) using a series of biochemical tests. Among all strains screened, HRRK 170 had the highest potential for plant growth promotion, given its ability to produce plant growth substances and enzymes such as siderophores and 1-aminocyclopropane-1-carboxylic acid (ACC) deaminase, respectively, concomitantly with active growth in a wider range of temperatures (10–30 °C) and pH (5.0–10.0). HRRK 170 colonized either as spots or widely on the root surface of all vegetable seedlings tested, but significant growth promotion occurred only in two vegetables (Chinese cabbage and green pepper) within a certain cell density range localized in the plant roots. The results indicate that HRRK 170 could function as a plant growth promoter, but has an optimum cell density for efficient use.

## 1. Introduction

Plants associate and form different relationships with diverse microorganisms in nature, including with plant growth-promoting bacteria that efficiently colonize plant roots. While root exudates fuel microbial growth and root tissues provide shelter [1], plant growth-promoting bacteria may accelerate plant growth at different stages via several mechanisms, either simultaneously or sequentially [2]. Today, intensive agriculture depends on excessive use of chemical fertilizers with known public health and environmental hazards [3,4,5]. Thus, environmentally friendly alternatives such as microorganisms have become attractive. Indeed, microbial bioinoculants may potentially reduce the use of chemicals while simultaneously enhancing crop productivity [5,6].

Plant growth-promoting bacteria may interfere with hormone production (e.g., auxin and cytokinin) and promote nutrient uptake (e.g., by solubilizing phosphate or by producing siderophores) [7,8]. Some bacteria may promote tolerance against phytopathogens by stimulating systematic resistance, producing hydrolytic enzymes that inhibit interactions with pathogens [3], and alleviating the effect of abiotic stressors by expression of a deaminase that hydrolyzes 1-aminocyclopropane-1-carboxylate (ACC), the immediate ethylene precursor [7,9]. Particularly, bacteria that fix nitrogen or solubilize minerals that are not bioavailable have been widely studied under various conditions and soil types for their use as commercial products [10,11], indicating that the mutual association with bacteria is one of the key factors determining plant health, productivity, and soil fertility [4,5,12].

The effectiveness of plant growth-promoting bioinoculants likely depends on bacterial tolerance to abiotic and biotic stress, which is necessary to sustain beneficial interactions with the host plant [8]. Furthermore, the efficiency of bioinoculants is determined by the ability to colonize roots and then to compete with other soil microorganisms and survive. Thus, bacterial adhesion is critical to prevent the loss of plant‒microbe interactions [13,14]. Various studies have investigated the mechanisms of efficient plant‒microbe interactions. Noirot-Gros et al. [15] reported that the formation of bacterial biofilms, in which microbial cells live in self-synthesized extracellular polymeric substances, resulted in different formations, dispersion, and colonization patterns, followed by plant growth-promoting efficiency. These results suggest that the effect of plant growth-promoting bacteria on plant growth is correlated with the number, dispersion, and colonization of infecting cells associated with their biofilm formations in plant tissues.

Plants are naturally exposed to numerous environmental stresses that affect growth, resulting in massive losses in production [16]. In the Tokachi area, Hokkaido, Japan, volcanic ash causes soil acidity and aluminum toxicity [17], which reduces crop yield by restricting root systems and the uptake of water and nutrients [18]. However, yields of sugar beet, which are important as both a source of sugar and in crop rotation, are persistently high, presumably because of the interactions with useful microorganisms such as plant growth-promoting bacteria [6]. Thus, we anticipated that bacterial strains isolated from the sugar beet may also increase the growth of other plants such as vegetables. Plant growth-promoting bacteria have been reported to mediate the growth promotion of vegetables caused by the increased nutrient availability and phytohormones regulation, resulting in high quality, safety, and freshness [10].

In a previous study, bacterial strains having a high affinity with the sugar beet (*Beta vulgaris* L. cv. Rycka) were examined for their plant growth-promotive abilities (Kenkyuseika, vol. 539. 2015. Tsukuba Office, Agriculture, Forestry and Fisheries Research Council Secretariat, Japan). In this study, we selected six potential plant growth-promoting bacterial strains (*Rhizobium* sp. HRRK 005, *Polaromonas* sp. HRRK 103, *Variovorax* sp. HRRK 170, *Mesorhizobium* sp. HRRK 190, *Streptomyces* sp. HRTK 192, and *Novosphingobium* sp. HRRK 193), and compared their biochemical characteristics in order to identify the strain with the highest ability of plant growth promotion. Then, the selected strain, *Variovorax* sp. HRRK170, was evaluated for its ability to promote plant growth using vegetable seedlings. The results demonstrate that HRRK170 is a plant growth promoter with beneficial biochemical properties, but has an optimum cell density for its full function.

## 2. Materials and Methods

### 2.1. Bacterial Strains and Growth Conditions

Bacterial strains with high affinity for sugar beet roots (*Beta vulgaris* L. cv. Rycka) [19] were previously studied for their plant growth promotion (Kenkyuseika, vol. 539. 2015. Tsukuba Office, Agriculture, Forestry and Fisheries Research Council Secretariat, Japan). Among them, six potential plant growth-promoting bacterial strains, *Rhizobium* sp. HRRK005 (NITE P-01604), *Polaromonas* sp. HRRK103 (NITE P-01607), *Variovorax* sp. HRRK170 (NITE P-01608), *Mesorhizobium* sp. HRRK190 (NITE P-01609), *Streptomyces* sp. HRTK192 (NITE P-01614), and *Novosphingobium* sp. HRRK193 (NITE P-01610), were used in this study. All strains were grown in R2A medium (BD, Sparks, MD, USA), and preculture was adjusted based on OD (OD_600_ = 0.1) to initiate culture. To determine growth at different temperatures and pH, a portion of preculture, grown aerobically in R2A medium at 30 °C, was transferred into the same fresh medium and grown at 10, 20, 30, and 40 °C or into the fresh medium adjusted to pH 4.0, 5.0, 6.0, 8.0, 9.0, and 10.0, and grown at 30 °C. Growth was monitored at 660 nm by using a biophotorecorder (TVS062CA; Advantec Co., Tokyo, Japan).

### 2.2. Biochemical Characteristics

Production of indole-3-acetic acid (IAA) was quantified by high performance liquid chromatography (HPLC) analysis using ethyl acetate extraction method [11]. In brief, bacterial cells were grown in 200 mL R2A broth containing 2 mM L-tryptophan at 30 °C and 130 rpm for 7 days. The supernatants were collected by centrifuging at 9800× *g* for 3 min, and after pH was adjusted to 2.8 by using 1N HCI, IAA was extracted with 200 mL ethyl acetate, twice. Then, the ethyl acetate fraction was evaporated under vacuum and the residue was dissolved using 1 mL methanol. Samples were filtered through a membrane filter (pore size, 0.2 µm) prior to HPLC analysis (Tosoh Co., Tokyo, Japan). IAA production levels were expressed as micrograms of IAA per mg dry weight of cells.

Siderophore production was evaluated using chrome azurol S shuttle assay [20] with minor modifications. In brief, bacterial precultures (50 µL) were inoculated into 5 mL of R2A broth and incubated at 30 °C and 130 rpm for 7 days. After centrifugation, 900 µL of the supernatant (*n* = 4) was incubated for 20 min with 100 µL chrome azurol S and 10 µL of 400 mM sulfosalicylic acid, the shuttle agent. As described above, the R2A broth (900 µL) reacted with chrome azurol S and was used as a reference. Siderophore production levels were expressed as the ratio of the final absorbance value of the sample [absorbance of the reference solution at OD_630_ minus absorbance of sample at OD_630_] against the reference solution.

Biofilm production was determined by microtiter plate assay [21], in which bacterial precultures adjusted to the same initial OD (OD_600_ = 0.1) were incubated statically at 20 °C for 48 h in 96-well polystyrene microplates. After removing the culture, the wells were air-dried and stained for 45 min with 200 µL of 1% crystal violet (FUJIFILM Wako Pure Chemical Co., Osaka, Japan). After dissolving in 95% ethanol, the product was assayed at 595 nm using a microplate reader (iMark, Bio-Rad Laboratories, Irvine, CA, USA).

Enzyme assays were conducted as described previously, but with slight modifications [22]. The 1-aminocyclopropane-1-carboxylic acid (ACC) deaminase activity was determined by measuring the amounts of α-ketobutyrate produced by the reaction. Briefly, bacterial cells were grown in R2A medium at 30 °C and 130 rpm for 48 h, and after centrifugation, washed with 5 mL of DF salts minimal medium [23]. Then, the cells were resuspended in 7.5 mL DF salts minimal medium containing 3 mM ACC and grown for 24 h to induce ACC deaminase activity. After the cells were washed with 1 mL of 0.1 M Tris-HCl (pH 7.6) and resuspended in 600 µL of 0.1 M Tris-HCl (pH 8.5), they were mixed with 30 µL toluene. Subsequently, the suspension (200 µL) was supplemented with 20 µL of 0.5 M ACC and 1 mL of 0.56 M HCl and further acidified with 800 µL of 0.56 M HCl, incubated at 30 °C for 15 min. Next, 300 µL of 0.2% 2,4-dinitrophenylhydrazine was added to the mixture and incubated at 30 °C for 30 min. The activity was expressed by the formation of α-ketobutyrate (nanomole per mg of cell for 1 h at given conditions).

β-1,3-glucanase activity was evaluated by measuring the amounts of reducing sugar released from laminarin. Briefly, bacterial cultures grown for 48 h in 1% colloidal chitin (Sigma-Aldrich, St. Louis, MI, USA) at 30 °C and 130 rpm were centrifuged at 4 °C and 9800× *g*, and 1 mL of the resulting supernatant was reacted at 40 °C for 1 h with 0.1 mL of 2% laminarin in 0.2 M acetate buffer (pH 5.4). Development of brown color was quantified at 530 nm. A standard curve was plotted using glucose, and one unit of the activity was defined as the amount of enzyme that liberates one micromole of glucose per hour at the indicated conditions.

Bacterial chitinase activity was measured on R2A agar plates supplemented with colloidal chitin [24], while cellulase activity was quantified on LB plates with carboxymethyl cellulose [25]. Lipase activity was assessed on R2A agar plates with Tween 20 (FUJIFILM Wako Pure Chemical Co., Osaka, Japan) as substrate [26]. These enzyme activities were quantified based on the diameter of halo zones formed around colonies after bacterial precultures (5 µL) were spotted on plates and incubated at 30 °C for 7 days.

### 2.3. Morphological Observation of HRRK170 by Scanning Electron Microscopy

HRRK170 colonies grown on R2A agar plates were visualized on a scanning electron microscope according to the conventional method. Briefly, cells grown at 30 °C for 48 h on R2A agar were smeared on a glass slide and, after immersion in 2% glutaraldehyde for 2 h, washed with 0.1 M phosphate buffer for 15 min, three times. Specimens were then treated with 50%, 75%, and 99.5% ethanol for 15 min, in this order, and immersed at 40 °C for 15 min three times in 99.0% tertiary butyl alcohol. Then, samples were lyophilized, coated with gold using an MSP-mini magnetron sputter (VD, Ibaraki, Japan), and imaged on a Miniscope TM3030 (Hitachi Hi-Tech Co., Tokyo, Japan).

### 2.4. Effect of HRRK170 on the Plant Growth

PotAce N potting soil (Katakura & Co-op Agri, Tokyo, Japan) was used to assess the plant growth. Vegetable seeds [cabbage (*Brassica oleracea* L. cv. Harunami), lettuce (*Lactuca sativa* L. cv. Cisco), tomato (*Solanum lycopersicum* L. cv. Momotaro), radish (*Raphanus raphanistrum* L. cv. Taibyo sobutori), eggplant (*Solanum melongena* L. cv. Senryo no.2), Chinese cabbage *(Brassica rapa* L. cv. Kigokoro 85, Kigokoro 65, Haregi 85, and Okiniiri), and green pepper (*Capsicum annuum* L. cv. Kyomidori, Kyonami, Ace, and Pitaro)] were purchased from Takii Seeds Co., Ltd. (Kyoto, Japan). Sugar beet seeds (*Beta vulgaris* L. cv. Rycka) were used as a reference and were provided by the Hokkaido Agricultural Research Center, National Agriculture and Food Research Organization, Hokkaido, Japan.

The vegetable seeds were sterilized with 70% ethanol for 1 min, 1% sodium hypochlorite for 1 min, and then rinsed with sterilized distilled water (three times). Radish and eggplant seeds were treated ultrasonically (200 W; US-105, SND Co., Ltd., Nagano, Japan) for 1 min (two times) before sterilization. Sugar beet seeds were left under running tap water for 24 h, sterilized with 70% ethanol for 1 min, 0.5% sodium hypochlorite containing 0.1% Tween 20 for 15 min, and then rinsed with sterilized distilled water for 15 min (three times). Sterilized seeds were sown on plug trays containing approximately 100 g soil and inoculated with 1 mL of 8.5 × 10^7^ CFU mL^−1^ HRRK170 cells per 4 seeds. Seeds were covered with aluminum foil for 7 days, and then grown for three weeks in a growth chamber (BiOTRON; NK system, Osaka, Japan) under cycles of 14 h light at 23.5 °C and 10 h dark at 20.0 °C. Plant parts above ground were weighed before and after drying at 60 °C for 3 days, to obtain the fresh and dry weights, respectively.

### 2.5. Tissue Localization of HRRK170 in Plant Roots

To observe plant tissue localization of HRRK170, a reporter gene, *gusA*, was introduced into the cells to construct GUS-labeled cells using methods described previously [27]. Briefly, *Escherichia coli* S17-1 harboring the plasmid pmTn*5*SS*gusA20* (donor) and HRRK 170 (recipient) cells were mixed, and after centrifugation, cell pellets were resuspended in 50 μL of 0.85% NaCl. Mating was performed at 30 °C for 2 days on a membrane filter (mixed cellulose ester; pore size 0.45 μm, Advantec Co., Tokyo, Japan) placed on R2A agar plate containing spectinomycin (100 μg mL^−1^), streptomycin (40 μg mL^−1^), and tetracycline (15 μg mL^−1^). Transformed HRRK170 cells were confirmed to promote plant growth to a similar extent as the wild type.

Sterilized seeds were sown on 1.5% agar plates and covered with aluminum foil prior to germination. Seedlings were then transferred to 0.3% agar plates containing a 2000-fold dilution of HYPONeX 6-10-5 (HYPONeX, Osaka, Japan), and after inoculation with 100 µL of GUS-labeled HRRK170 cells (8.5 × 10^7^ CFU mL^−1^) per seedling, grown in a growth chamber (BiOTRON; NK system, Osaka, Japan) under the same conditions described above. HRRK170 cells localized in seven-day-old seedlings were stained in a GUS-staining solution containing 16 mL of 125 mM sodium phosphate buffer (pH 7.0), 80 μL of 0.5 M Na_2_EDTA (pH 8.0), 200 μL of 2% 5-bromo-4-chloro-3-indolyl-β-d-glucuronide cyclohexylammonium salt, 80 μL of 10% sodium dodecyl sulfate, and 23.6 mL of distilled water. The staining was performed in de-aired conditions for 90 min and then left at 30 °C for 2 h prior to use. Microscopic observation of the localized HRRK170 cells in seedlings was conducted using a stereomicroscope (SZX16, Olympus Co., Tokyo, Japan). In parallel, agar (5%)-embedded sections of roots were sliced using a microslicer (DTK1000 ZERO1, Dosaka EM Co., Ltd, Kyoto, Japan) and the localization of cells inside the plant tissues was observed under an inverted microscope (BZ-X700, Keyence Co., Osaka, Japan).

### 2.6. Calculation of HRRK170 Cell Density Using Color Development by GUS Staining

To quantify HRRK170 cell density by color development of GUS-staining solution, the correlation between HRRK170 cell density and the absorbance value (OD_615_) of GUS-staining solution was examined. GUS-labeled HRRK170 cells were grown at 30 °C and 130 rpm, overnight in R2A broth containing the antibiotics described above, and serially diluted cell suspensions were prepared. After 2 mL of each diluted cell suspension was centrifuged at 4 °C and 18,000× *g* for 5 min, the resultant cell pellets were suspended in 2 mL GUS-staining solution and stained as described above. After removing cells by centrifugation, the absorbance (OD_615_) of the supernatant was measured using a spectrophotometer (Ultrospec 3100 pro, GE Healthcare Life Sciences, Buckinghamshire, UK). In parallel, the cell number involved in each diluted cell suspension was also determined using the plate dilution method to make a correlogram with the OD_615_ values of GUS-staining solution. To calculate the plant-infected cell density of HRRK170, 10 seedlings inoculated with GUS-labeled HRRK170 cells were mixed with 20 mL of GUS-staining solution, and the absorbance (OD_615_) of the solution was measured. Then, the cell number was calculated using the correlogram, and cell density was expressed as the infected cell number per g weight of plant.

### 2.7. Statistical Analysis

The statistical analysis was performed using IBM SPSS Statistics for Windows v.23.0 (IBM, Armonk, NY, USA). Data were subjected to the Student’s *t*-test. A Tukey’s honestly significant difference test with post-hoc comparison at the 5% confidence level was used to compare mean values among treatments. Experiments were performed with at least three replicates for each treatment.

## 3. Results

### 3.1. Bacterial Growth Under Different Temperatures and pH Values

**Figure 1:**
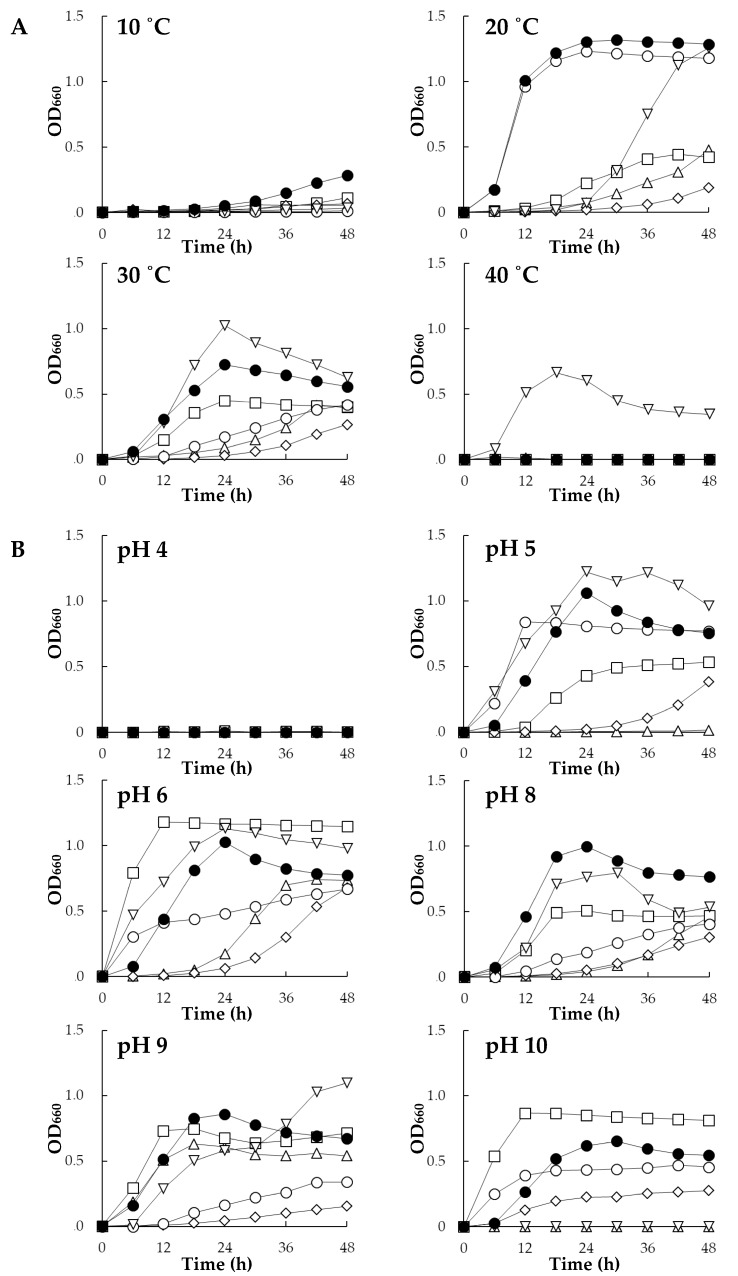
Growth of six plant growth-promoting bacteria under different temperatures (**A**) and pH values (**B**). Growth was monitored at OD_660_ using a biophotorecorder. Data are the means of three replicates. Symbols: 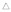, HRRK005; □, HRRK103; ●, HRRK170; ◊, HRRK190; 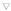, HRTK192; ○, HRRK193.

Figure 1 shows growth profiles of the six strains (*Rhizobium* sp. HRRK005, *Polaromonas* sp. HRRK103, *Variovorax* sp. HRRK170, *Mesorhizobium* sp. HRRK190, *Streptomyces* sp. HRTK192 and *Novosphingobium* sp. HRRK193) under different temperatures and pH ranges. All bacterial strains grew at 20 and 30 °C. HRRK170 showed the most active growth at both temperatures with a growth peak at 24 h after incubation. In addition, HRRK170 also showed the highest growth at 10 °C (Figure 1A). Only HRTK192 grew at 40 °C, but showed a lag time of 24 h at 20 °C. The growth of HRRK193 was active at 20 °C and close to that of HRRK170. However, its growth at 30 °C was considerably slower than that of HRRK170 and HRTK192. The other strains, HRRK005, 103, and 190, showed similar growth profiles and their growth was slower at both 20 and 30 °C, although HRRK103 grew slowly at 10 °C (Figure 1A). For pH, all strains grew at pH 6.0 to 9.0. Among them, HRRK170 exhibited active growth in a wide range of pH values (5.0 to 10.0) with a growth peak at approximately 24 h. HRTK192 also exhibited active growth from pH 5.0 to 9.0, which was close to that of HRRK170 at 24 h after incubation. However, growth was inhibited at pH 10.0. HRRK103 showed the highest growth at pH 6.0 and 10.0, but growth at other pH values was lower than that of HRRK170 at 24 h after incubation. Growth of HRRK005, 190, and 193 was slower than that of these three strains at pH 6.0 to 8.0, and HRRK005 showed no growth at pH 5.0 and 10.0. These results indicated that HRRK170 had the highest ability to grow in a wider range of temperatures (10 to 30 °C) and pH values (5.0 to 10.0) among the six strains.

### 3.2. Biochemical Characteristics of Six Strains

Based on the growth profiles of six strains under different temperatures and pH values as shown in Figure 1, we investigated their biochemical activities in order to evaluate their abilities to produce plant growth substances and enzymes at 30 °C (20 °C for biofilm production) and a neutral pH (Table 1). All six strains produced siderophores, biofilm, and IAA. Particularly, HRRK170 exhibited higher production of these compounds and the production levels of siderophores and biofilm were the highest among the six strains (52.63 and 0.46, respectively) (Table 1). Regarding enzyme production, activity of ACC deaminase and β-1,3-glucanase was also observed in almost all of the six strains. However, the level of ACC deaminase produced by HRRK170 was significantly higher than that of the other strains, and the level reached approximately 60.41 (nmol AKB mg^−1^ cell h^−1^) (*p* < 0.001) (Table 1). Both cellulase and lipase activities were detected in HRRK005, HRRK170, and HRTK192, but chitinase activity was detected only in HRRK005 (Table 1). Results obtained from the biochemical analyses as well as the growth profile indicated that HRRK170 had the highest potential as a plant growth promoter.

### 3.3. Morphological Characteristics of HRRK170

**Figure 2:**
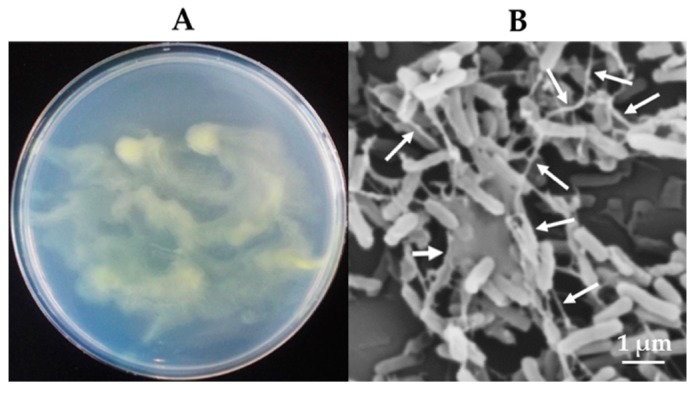
Morphological characteristics of *Variovorax* sp. HRRK170 cells. (**A**) Cells grown on R2A agar at 30 °C for 48 h. (**B**) Microscopic observation of HRRK 170 cells grown on R2A agar using a scanning electron microscope. Biofilms produced by HRRK 170 are indicated by the arrows.

Figure 2 shows morphological characteristics of *Variovorax* sp. HRRK170 grown on an R2A agar plate. HRRK170 cells grown on R2A agar were gelatinous, glistening, yellowish, and robustly proliferative. However, the colony shape was obscure and smeary (Figure 2A). In addition, cells were straight or slightly curved rods, approximately 1.5 µm long, and embedded in a mucilaginous layer due to its ability to produce biofilm, as visualized using scanning electron microscopy (Figure 2B).

### 3.4. Effect of HRRK170 on the Growth of Vegetable Seedlings

**Figure 3:**
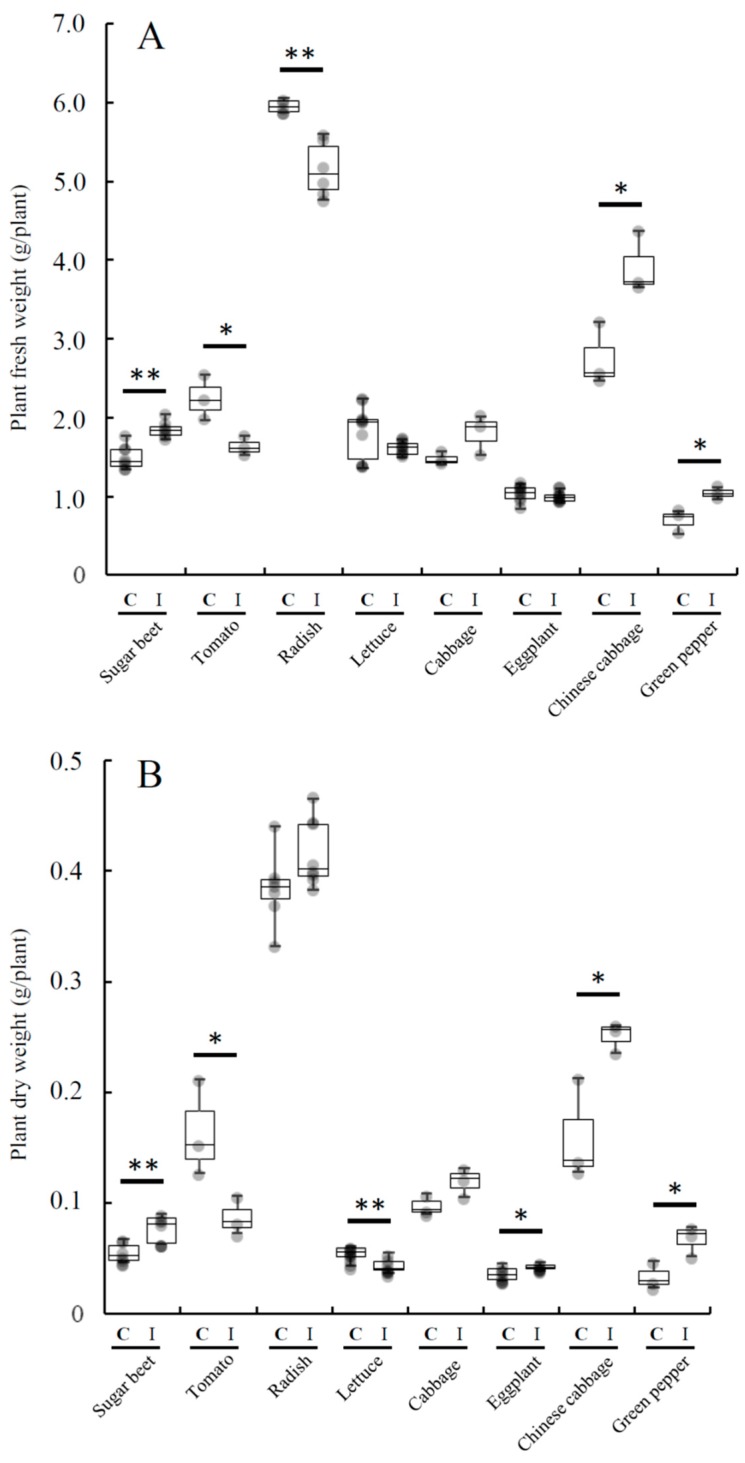
Effect of *Variovorax* sp. HRRK170 on the growth of vegetable seedlings. Plant fresh weights (**A**) and dry weights (**B**) are shown as means ± standard deviation. Mean values were compared by t-test. Asterisks indicate significant (*, *p* < 0.05; **, *p* < 0.01) differences between inoculated (I) and uninoculated control (C). Host plants used: tomato cv. Momotaro, radish cv. Taibyo sobutori, lettuce cv. Cisco, cabbage cv. Harunami, eggplant cv. Senryo 2 go, Chinese cabbage cv. Haregi 85, green pepper cv. Kyonami, and sugar beet cv. Rycka as a reference.

Figure 3 shows the inoculation effect of *Variovorax* sp. HRRK170 on the growth of seven vegetable seedlings in addition to sugar beet, which was used as a reference. The results showed that HRRK170 significantly promoted the growth of Chinese cabbage and green pepper as well as sugar beet compared with the uninoculated control (ratio (%) of the weights of inoculated plants against those of uninoculated plants: Chinese cabbage, 142.4 and 157.1; green pepper, 149.0 and 200.2; sugar beet, 124.1 and 137.8, in terms of fresh and dry weights, respectively). The growth of eggplant also significantly increased with the inoculation of HRRK170 in terms of dry weights (ratio (%) against the uninoculated control: 118.9). In contrast, the growth of tomato and lettuce significantly decreased in terms of dry weights, particularly that of tomato, which was significantly decreased with the inoculation in terms of both fresh and dry weights (ratio (%) against the uninoculated control: 72.5 and 53.0, in fresh and dry weights, respectively).

### 3.5. Plant Tissue Localization of HRRK 170

**Figure 4:**
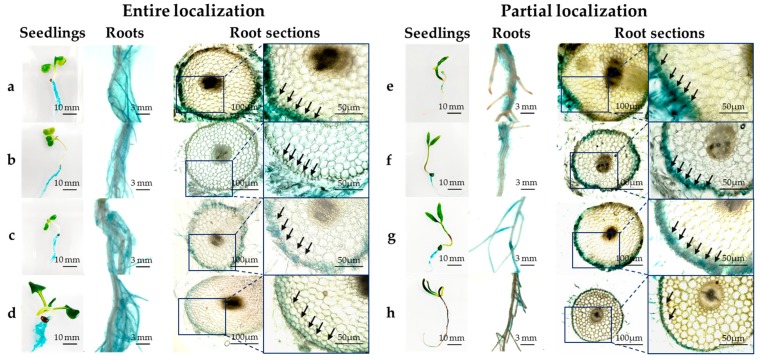
Tissue localization of *Variovorax* sp. HRRK170 in seven vegetable seedlings and sugar beet as a reference. The cells localized in the seedlings at 7 days post-inoculation were detected using GUS staining, and entire (left panels) and partial (right panels) localization was observed using a stereomicroscope. (**a**) Chinese cabbage (cv. Haregi 85), (**b**) cabbage (cv. Harunami), (**c**) lettuce (cv. Cisco), (**d**) radish (cv. Taibyo sobutori), (**e**) green pepper (cv. Kyonami), (**f**) eggplant (cv. Senryo 2 go), (**g**) tomato (cv. Momotaro), and (**h**) sugar beet (cv. Rycka) as a reference. Boxes show the enlarged regions in insets, and arrows indicate the localization of HRRK 170 cells. Photos of seedlings, roots, and root sections were obtained using a digital camera, stereomicroscope, and inverted microscope, respectively.

Figure 4 shows localization of HRRK170 in plant tissue during its initial interaction with the seven vegetable seedlings and the sugar beet as a reference, 7 days post-inoculation. HRRK170 colonized all plants by 7 days post-inoculation, but was localized only in plant roots as shown in GUS-stained plant tissues. In addition, the localization profiles of HRRK170 in roots were divided into two types: entire and partial. The colonization throughout the roots was observed in Chinese cabbage (a), cabbage (b), lettuce (c), and radish (d), whereas partial but prominent colonization in the roots was in green pepper (spot-like colonization) (e), eggplant (uneven colonization) (f), tomato (uneven colonization) (g), and sugar beet (colonization including root hairs) (h) (Figure 4). Next, the transverse root sections were prepared and internal infection of HRRK170 into the plant tissue was assessed using GUS staining. Results indicated that HRRK170 localized to the epidermis, including root hairs, despite the localization profiles at 7 days post-inoculation (Figure 4).

### 3.6. Evaluation of HRRK 170 Cell Density Localized in the Plant Roots

**Figure 5:**
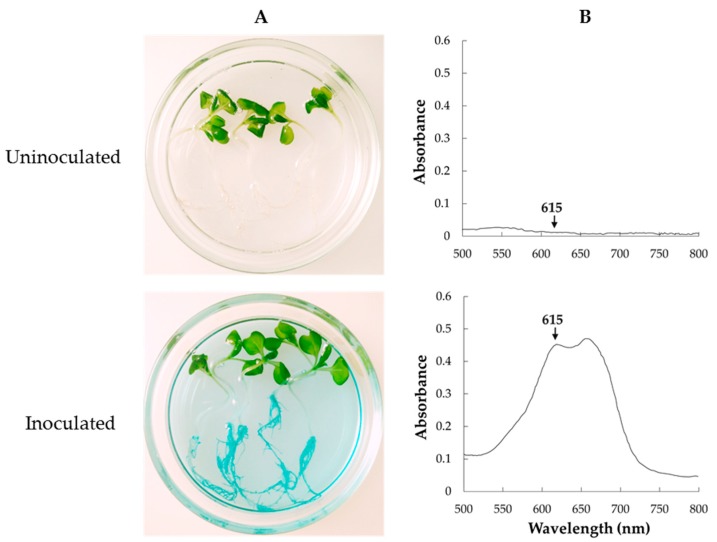
Color development in the reaction solution of GUS-stained *Variovorax* sp. HRRK 170 localized in the roots (Chinese cabbage, cv. Haregi 85) (**A**) and an absorption wavelength of the color-developed reaction solution (**B**). The absorbance of indigo-blue chromophore produced by the enzymatic hydrolysis of β-glucuronidase (OD_615_) is shown by the arrows.

Since HRRK 170 promotes or represses plant growth, as shown in Figure 3, we tested the possibility that the cell density localized in the plant roots may explain this contradictory effect. Figure 5A shows the color development of GUS-staining solution using GUS-marked HRRK170 localized in the roots. This finding prompted us to estimate the cell density using the staining intensity because this method is faster and more convenient than the agar plate dilution method, which is generally used to count cell number.

**Figure 6:**
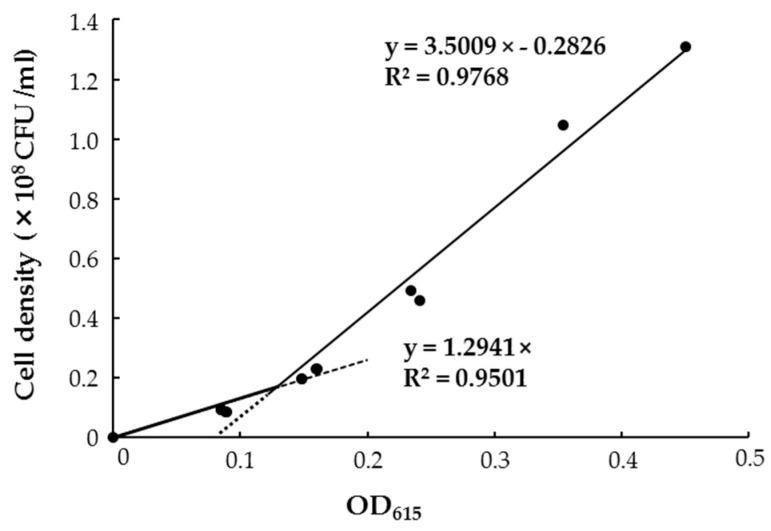
Correlation between *Variovorax* sp. HRRK 170 cell density and absorbance value (OD_615_) of the reaction solution colored using GUS staining.

To subject the staining intensity to colorimetry, the most appropriate wavelength in absorption spectra was first obtained from the color-developed solution caused by GUS-stained HRRK170 cells, i.e., absorbance at 615 nm was used as an index of staining intensity (Figure 5B). Then, the correlation between the cell density of HRRK170 (cell number per mL) and OD_615_ was assessed (Figure 6). Interestingly, the result indicated that OD_615_ was linearly correlated with the cell density obtained from plate dilution method, although absorbance data below or above 0.128 were fitted to different correlation coefficients in order to achieve better linearity (Figure 6). The cell densities were then estimated using OD_615_ with this correlation curve.

**Figure 7:**
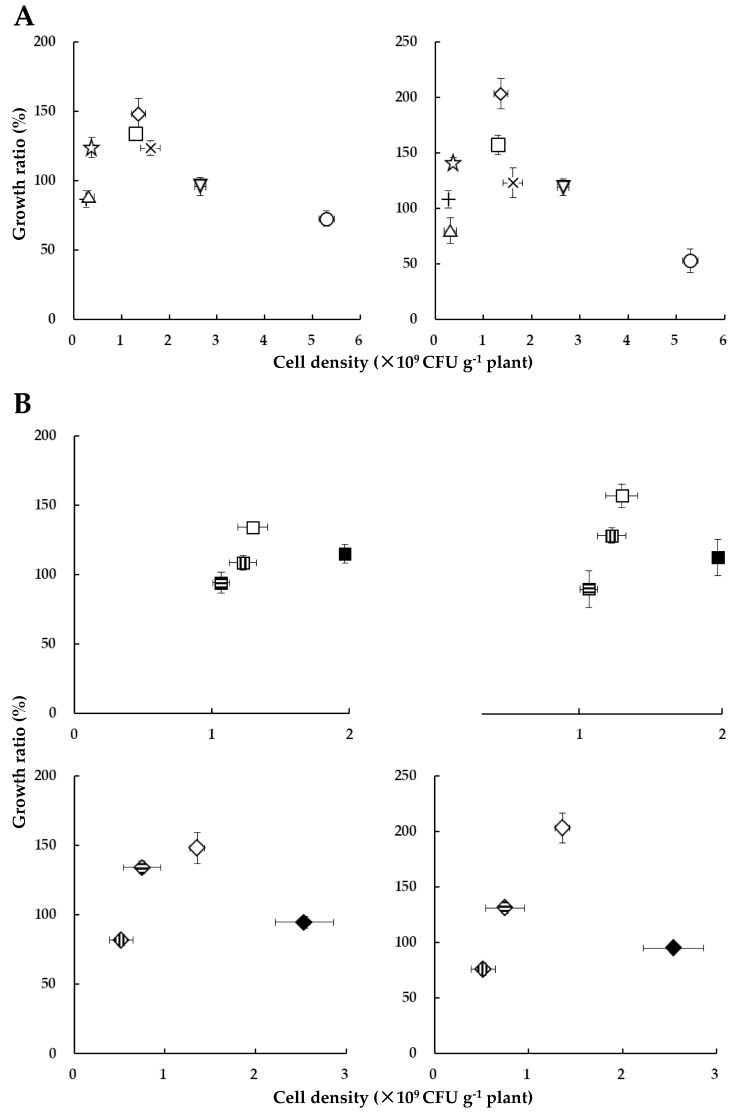
Relationship between *Variovorax* sp. HRRK 170 cell density and growth ratio (%) of seedlings for seven vegetables, with sugar beet as a reference (**A**) and for the four cultivars of both Chinese cabbage and green pepper (**B**). Vertical axes show the ratio (%) of the plant weight inoculated with HRRK 170 to that without it (growth ratio for fresh and dry weights is shown on the left and right, respectively). Cell density was calculated as the cell number per g of plant using the correlation curve described in Figure 6. Vertical and horizontal bars show means ± standard deviation. Symbols used (**A**): 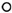, tomato cv. Momotaro; 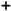, radish cv. Taibyo sobutori; 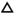, lettuce cv. Cisco; 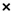, cabbage cv. Harunami; 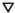, eggplant cv. Senryo 2 go; 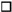, Chinese cabbage cv. Haregi 85; 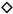, green pepper cv. Kyonami; 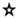, sugar beet cv. Rycka as a reference. (**B**): Chinese cabbage: 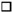, cv. Haregi 85; 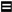, cv. Kigokoro 65; 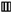, cv. Kigokoro 85; 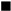, cv. Okiniiri. Green pepper: 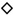, cv. Kyonami; 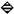, cv. Kyomidori; 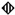, cv. Ace; 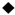, cv. Pitaro.

Figure 7A shows the relationship between cell density of HRRK170 (cell number per g of plant) and the plant growth ratio (percent of the inoculated plant weights against uninoculated control, which was calculated from the plant weights obtained in Figure 3) for seven vegetables with sugar beet as a reference. Results indicated that the plant growth ratio (%) was closely related to the cell density and had its optimum values for the plant growth promotion, i.e., green pepper and Chinese cabbage exhibited higher values of both growth ratio and cell density than radish and lettuce, and their growth ratios were significantly higher than those of the other five vegetables (Figure 3 and Figure 7A). Interestingly, the cell densities of cabbage, eggplant, and tomato were higher, while their growth ratios were lower than those of green pepper and Chinese cabbage. Notably, the growth ratio of tomato was considerably reduced, although it was infected with the highest number of cells.

Next, we sought to determine whether the relationship between the plant growth ratio and cell density would also be present in several cultivars of the same host plant. Therefore, we used four cultivars of Chinese cabbage and four of green pepper. Interestingly, a similar relationship was found in these cultivars, i.e., the plant growth ratio reflected a cell density-dependent manner with its optimum values (Figure 7B). Particularly, for green pepper, the growth ratio was increased with an increase in the cell density for the three cultivars (Ace, Kyomidori, and Kyonami, in this order), but decreased for the cultivar Pitaro, for which the cell density was the highest among the four cultivars (Figure 7B). These results indicated that HRRK170 promoted plant growth within a certain range of cell densities, i.e., at an optimum cell density for its full function in the early interaction with host plants.

## 4. Discussion

In this study, we screened six potential plant growth-promoting bacterial strains, which were previously isolated from sugar beet, and selected *Variovorax* sp. HRRK170 for having the highest ability to produce plant growth substances and enzymes such as siderophores and ACC deaminase, respectively (Table 1). HRRK170 also showed higher tolerance against lower temperature stress (10 °C) (Figure 1). In addition, biofilm production was evaluated at 20 °C, 48 h, because our preliminary experiment showed that a higher cell number was suited to the production of biofilms for the strains (Figure 1). The results showed that this strain also had a higher ability to produce biofilms (Table 1 and Figure 2). The type species, *V. paradoxus*, is known to have straight to slightly curved rods, and the colonies on nutrient agar are normally convex, glistening, shiny, and yellow or greenish yellow in color. Additionally, growth factors are not required because of the availability of a wide variety of organic compounds [28]. Genomic analyses by Han et al. revealed that *V. paradoxus* S110 was metabolically diverse and highly adaptable to various environmental conditions, and thus may support plant growth and degrade toxic compounds. This species may also promote plant growth by recruiting other bacteria that convert various substrates into cell biomass [29]. Belimov et al. reported that nitrogen accumulated in soils planted with seeds that were inoculated with *V. paradoxus* 5C-2 [9]. These results suggest that HRRK170 could be one of the most promising plant growth-promoting bioinoculants.

Notably, HRRK170 colonized the roots of all seven vegetable seedlings, as well as the sugar beet, seven days after inoculation and significantly promoted the growth of two vegetable seedlings (Chinese cabbage and green pepper) as well as the sugar beet, and also showed an upward tendency for two vegetable seedlings (cabbage and eggplant) (Figure 3 and Figure 4). However, HRRK170 failed to function as a plant growth promoter for the other three vegetables (tomato, radish, and lettuce) (Figure 3). The colonization profiles of HRRK170 showed two different types (entire and partial) but did not seem to be correlated with the inoculation effects of HRRK170 (Figure 3 and Figure 4). To elucidate the contradictory behavior of HRRK170, the number of HRRK170 cells infecting each plant was estimated in order to evaluate the relationship with plant growth promotion. Typically, cell numbers of the infecting strain are calculated via the plate dilution method after crushing plant tissues. However, since GUS-stained HRRK170 cells were found to cause color development due to GUS-staining solution, the cell number was estimated by the absorbance value (OD_615_) because this method provided more accurate information after only 3.5 h incubation without crushing the plant tissues. The substrate, 5-bromo-4-chloro-3-indolyl-β-d-glucuronic acid (X-Gluc), for β-glucuronidase produces an insoluble, intense, indigo-blue chromophore (OD_615_) after enzymatic hydrolysis [30]. Interestingly, the color development of the reaction solution is based on the production of this indigo-blue chromophore caused by enzymatic hydrolysis of X-Gluc, and its absorbance value (OD_615_), which is less than approximately 0.45, correlated with the cell number, indicating that it could be estimated using this absorbance value (Figure 6). However, it remains unclear why this correlation curve does not show a simple linearity, since it is considered that the *gusA* gene encoding ß-glucuronidase is constitutively expressed from the promoter [31]. It may also be possible that the *gusA* expression was affected by cell number in such a way as to be lowered under higher cell number.

The cell density of HRRK170 localized in the plant roots (cell number per g of plant) could be estimated using this correlation curve because most of the cells localized on the epidermis despite the localization profiles at 7 days post-inoculation (Figure 4). This study showed that *Variovorax* sp. HRRK 170 promoted plant growth within a certain range of cell densities, indicating that it has an optimum cell density for its full function. Excess cell density may lose symbiotic balance with the host plant, whereas low affinity with the host plant results in low density of infecting cells. The complexity of beneficial plant‒microbe interactions associated with cell number of microbes is still being discovered. Several studies have investigated the effects of initial inoculant concentration on plant growth. Larcher et al. reported that the initial dose of bacterial inoculant regulated the length of rapeseed root and shoot, suggesting that the cell number affects plant growth promotion [32]. Suckstorff et al. also reported that *Stenotrophomonas* sp. showed plant growth-promoting behavior in a dose-dependent manner [33]. In addition, efficient plant tissue localization of inoculants sustains plant growth in such a way as to increase the competitiveness against other soil microorganisms or to prevent the infection of harmful pathogens [22]. However, our results indicate that infecting cell density is an important factor in determining whether or not the bacterial strain fully functions to promote plant growth, and optimum cell density must be considered for the application of plant growth-promoting bioinoculants to host plants. Besides, prompt counting of cell number based on GUS-staining methodology would simplify the evaluation of infecting cell density in plants and provide new insight into plant‒microbe interactions. Further studies are needed to clarify how the infecting cell density affects plant growth promotion.

## Figures and Tables

**Table 1:** Biochemical activities of six plant growth—promoting bacteria.

Strain	Plant Growth-Promoting Compounds and Enzyme Activities							
	Siderophore (%)	Biofilm (OD_595_)	IAA (µg mg^−1^ Dry cell)	ACC Deaminase (nmol AKB mg^−1^ Cell h^−1^)	β-1,3-Glucanase (U mL^−1^)	Cellulase (mm dia.)	Lipase (mm dia.)	Chitinase (mm dia.)
HRRK005	17.87 ± 0.23^b^	0.26 ± 0.01^a^	92.55 ± 0.55^c^	n.d.	0.02 ± 0.03^a^	0.35 ± 0.15^a^	1.34 ± 0.56^b^	0.50 ± 0.08
HRRK103	13.14 ± 1.63^a^	0.19 ± 0.02^a^	74.31 ± 1.58^b^	0.98 ± 0.03^a^	4.24 ± 0.10^d^	n.d.	0.13 ± 0.42^a^	n.d
HRRK170	52.63 ± 1.70^c^	0.46 ± 0.09^b^	100.52 ± 2.02^d^	60.41 ± 2.47^d^	0.62 ± 0.15^b^	0.43 ± 0.25^a^	3.24 ± 0.83^c^	n.d
HRRK190	13.07 ± 3.02^a^	0.25 ± 0.03^a^	92.54 ± 3.79^c^	5.86 ± 0.50^b^	0.67 ± 0.30^b^	n.d.	n.d.	n.d
HRTK192	10.77 ± 1.45^a^	0.43 ± 0.06^b^	118.00 ± 0.82^e^	20.74 ± 1.92^c^	0.46 ± 0.11^b^	2.24 ± 0.40^b^	3.36 ± 0.66^c^	n.d
HRRK193	11.99 ± 2.32^a^	0.44 ± 0.09^b^	16.43 ± 0.42^a^	19.58 ± 0.00^c^	1.46 ± 0.11^c^	n.d.	n.d.	n.d

Values are means ± standard deviation. The significance of differences between means was compared using one-way analysis of variance. Mean values in the same column with common superscript letters are not significantly different (*p* ≤ 0.05) by Tukey’s honestly significant difference test. n.d.: not detected.
